# Electrochemical Investigations of Steels in Seawater Sea Sand Concrete Environments

**DOI:** 10.3390/ma14195713

**Published:** 2021-09-30

**Authors:** Xiang Yu, Saad Al-Saadi, Xiao-Ling Zhao, R. K. Singh Raman

**Affiliations:** 1 Department of Chemical Engineering, Monash University, Clayton, VIC 3800, Australia; xiang.yu@monash.edu; 2 Department of Mechanical and Aerospace Engineering, Monash University, Clayton, VIC 3800, Australia; saad.al-saadi@monash.edu; 3 School of Civil and Environmental Engineering, University of New South Wales, Sydney, NSW 2052, Australia; xiaolin.zhao@unsw.edu.au

**Keywords:** seawater and sea sand concrete (SWSSC), open circuit potential (OCP), potentiodynamic polarization (PDP), electrochemical impedance spectroscopy (EIS)

## Abstract

Seawater and sea sand concrete (SWSSC) is an environmentally friendly alternative to ordinary Portland cement concrete for civil construction. However, the detrimental effect of high chloride content of SWSSC on the corrosion resistance of steel reinforcement is a concern. This study undertook the electrochemical corrosion behaviour and surface characterizations of a mild steel and two stainless steels (AISI type 304 and 316) in various simulated concrete environments, including the alkaline + chloride environment (i.e., SWSSC). Open circuit potential (OCP), potentiodynamic polarization (PDP), electrochemical impedance spectroscopy (EIS) and scanning electron microscopy (SEM) were employed. Though chloride is detrimental to the corrosion resistance of mild steels, a simultaneous presence of high alkalinity in SWSSC negate the detrimental effect of chloride. In the case of stainless steels, a high level of alkalinity is found to be detrimental, whereas chloride seems to have less detrimental effect on their corrosion resistance.

## 1. Introduction

In response to the shortage of resources caused by huge increase in demand for infrastructure and hence construction materials in the large-scale industrial world, seawater and sea sand concrete (SWSSC) is an attractive alternative to the ordinary Portland cement (OPC) concrete [1,2,3,4,5]. SWSSC is deemed as a new concrete type, which uses seawater to replace fresh water, and sea sand to replace river sand [6,7]. The design of SWSSC can reduce the over-use of fresh water and river sand. Because the mining of river sand for use in concrete aggregate has a negative impact on river ecosystems, navigation and flood control, SWSSC will be beneficial to the environment [6]. According to [8,9], the mechanical properties of SWSSC are similar to OPC. SWSSC can be further improved by using industrial waste, including alkali-activated slag, fly ash as a binding material [8,10,11]. The concrete that uses such materials (called high performance concrete (HPC)) require lesser use of cement, thereby has great environmental benefits since OPC production is highly energy-intensive and contributes considerably to carbon dioxide emissions [1]. Accordingly, SWSSC can be sea water and sea sand normal concrete (SWSSNC) that uses Portland cement, or sea water and sea sand high performance concrete (SWSSHPC) that uses fly ash or slag. Based on the research of the Japan Concrete Research Institute [12], concrete produced using seawater and slag tends to possess higher strength than that of concrete produced using tap water. In addition, the alkali-silica reaction has an adverse effect on ordinary Portland cement concrete, further destroying the integrity of the structure [13]. The utilization of SWSSHPC can reduce the probability of concrete spalling and cracking caused by alkali silica reactions (ASR) [10,11].

Generally, low-carbon steel or mild steel rebar is used as a reinforcing material for concrete to obtain high tensile strength and ductility. Nevertheless, carbon steel may be susceptible to prohibitively high corrosion rates due the high chloride content of seawater in SWSSC [10,14,15]. It is known that mild steel rebar can form a protective passivation layer in the strong alkaline environment of concrete (when pH > 10) [15]. However, this layer can be destroyed by chloride ions and other harmful ions, diffusing from the ocean or industrial environment through the concrete matrix to the surface of the steel bars, causing rapid corrosion of the steel bars. Such rapid corrosion of steel rebar can cause concrete cracking and spalling, which is called “concrete cancer”, that seriously jeopardizes the durability of concrete structures. Consequently, the premature failure of structures caused by steel bar corrosion is considered to be a key durability issue, even for OPC [15,16,17]. This deterioration of reinforced concrete leads to high repair and maintenance costs [18]. Therefore, it should be considered whether it is necessary to adopt steels with greater resistance to chloride-assisted corrosion, such as stainless steel (SS), to replace low-carbon steels in aggressive environments such as SWSSC. Austenitic stainless steels, AISI316 (SS316) and AISI304 (SS304) are the most commonly used stainless steels and are endowed with good corrosion resistance because they have a very thin, strong, self-healing and protective nickel and chromium enriched oxide layer [19,20]. Chromium oxide accumulates inside to form a barrier layer of oxide film, while the oxides of iron accumulate on the outside of the passive film, described as an exchange layer that is in contact with the environment [21]. SS316 contains approximate 2% molybdenum, which can improve the pitting resistance in aggressive aqueous environments (such as seawater and deicing salt) [22,23]. Molybdenum plays several beneficial roles in the corrosion resistance of SS [24,25]. Cramer et al. [26] also asserted that the use of SS, instead of low-carbon steel reinforcement, can reduce maintenance costs by more than 50%, and the expected service life of OPC far exceeds 100 years. Nevertheless, if the steels are exposed to an environment with extremely high chloride concentration, it may still damage the chromium oxide protective layer of SS, which compromises their corrosion resistance and results in localized corrosion, such as pitting [27,28,29].

There has been considerable research on the corrosion resistance of mild steel and SS in individual alkaline and aqueous chloride environments. It is found that the destruction of the passivation film is caused by Cl^−^ ions absorbed by the passivation film that can penetrate the film and weaken the binding between the passivation layer and the metal [30,31]. For stainless steels, the passive film breaks down in salt water more quickly at lower chromium contents [32]. In the alkaline environment, the passive film formed on the steel has a multilayer structure, with an inner layer of Fe_3_O_4_ and an outer layer of iron hydroxide [32,33]. The understanding of corrosion resistance of mild steel and SS in a mixed alkali + chloride environment (such as SWSSC) is extremely limited. Another area with little information in the literature is the role of seawater in the corrosion of stainless steel in high-performance concrete (i.e., SWSSHPC) when compared with SWSSNC environment. Consequently, this study will focus on the corrosion resistance of a mild steel and two SS in various concrete environments.

This study investigates the electrochemical corrosion behaviour of a mild steel and the two most common stainless steels, 316 and 304, in simulated solutions of seawater, concrete and seawater sea sand concrete (SWSSC) environments. Open circuit potential (OCP) measurement and electrochemical impedance spectroscopy (EIS) were employed for the three steels in the simulated solutions of normal concrete (NC), seawater sea sand normal concrete (SWSSNC), high performance concrete (HPC), and seawater sea sand high performance (SWSSHP) for up to 168 h. Simulated seawater (SW) was used for comparison. Potentiodynamic polarization tests (PDP) were conducted to investigate the passivation behaviour. Post-corrosion surface topography was characterized using scanning electron microscopy (SEM).

## 2. Materials and Methods

### 2.1. Sample Preparation and Test Solutions

Coupons of stainless steels (SS316 and SS304) with dimensions of (20 mm × 20 mm × 12 mm) were cut from steel strips purchased from George Archer Metals, Australia. The mild steel coupons were cut from steel strips procured from BlueScope Steel, Australia. Table 1 shows the chemical compositions of the steels. Prior to electrochemical tests, the steel coupons were ground with SiC papers (180 to 2500 grit size), ultrasonically cleaned with acetone and ethanol (10 min for each step), rinsed with DI water, and dried using compressed air.

Table 2 shows the five test solutions used in the present study, viz., those simulating NC, SWSSNC, HPC, SWSSHPC and SW. Simulated solutions of NC and HPC are prepared according to [34]. The simulated solutions of SWSSNC and SWSSHPC were prepared according to [4,15,35,36].

### 2.2. Electrochemical Methods

Open circuit potential (OCP), electrochemical impedance spectroscopy (EIS) and potentiodynamic polarization (PDP) were carried out using a VMP3 multi-channel potentiostat (Make: BioLogic, France) and a three-electrode electrochemical cell. The clean steel specimens (as described in Section 2.1) were used as the working electrodes, the saturated calomel electrode (SCE) was used as the reference electrode and a platinum mesh was used as the counter electrode. For EIS and PDP experiments, a defined area of the specimen was exposed to the test solution through a wide orifice in the corrosion cell. The specimen was held in the same position for the entire test duration (i.e., a maximum of 168 h) while the EIS measurements were carried out after the desired intervals. All the measurements were repeated at least three times to examine reproducibility. OCP was monitored and recorded for 168 h for all steels in different simulated solutions (Table 1). EIS was performed after different times of immersion in simulated solutions by applying a sinusoidal potential perturbation 10 mV at OCP. The impedance was measured at frequencies between 1 MHz and 10 mHz, recording 10 points per decade of frequency. PDP tests for the steel samples exposed to 168 h of different simulated solutions were carried out at a scan rate of 0.116 mV/s, starting at a potential of 250 mV more negative to the OCP.

### 2.3. Surface Characterization

A Phenom XL (ThermoFisher Scientific, Brisbane, QLD, Australia) desktop scanning electron microscope was used to examine the surface morphology after exposure to the different simulated solutions for 3, 72 and 168 h. At the end of each period of immersion, the steel samples were taken out of solution, immersed in DI water for a short period to remove the remaining test solution, dried with hot air stream and taken directly for SEM.

## 3. Results and Discussion

### 3.1. Open Circuit Potential (OCP)

**Figure 1:**
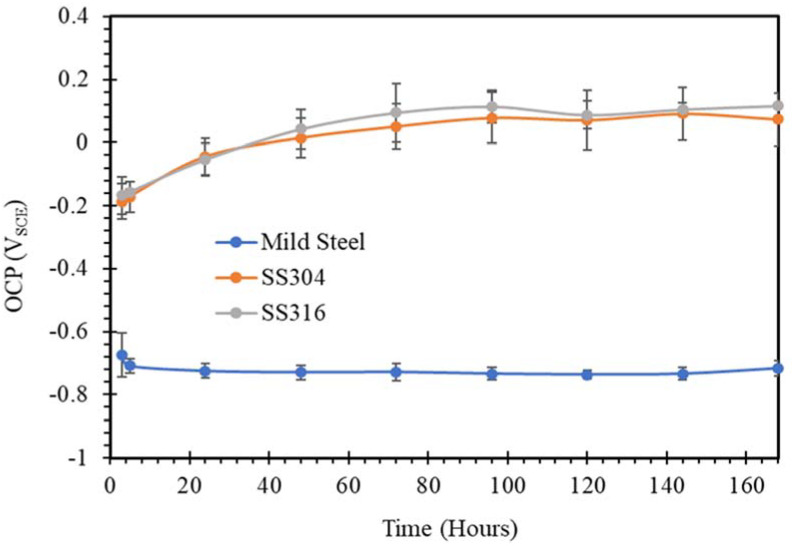
OCP vs. time plots of mild steel, SS304 and SS316 in SW solution.

**Figure 2:**
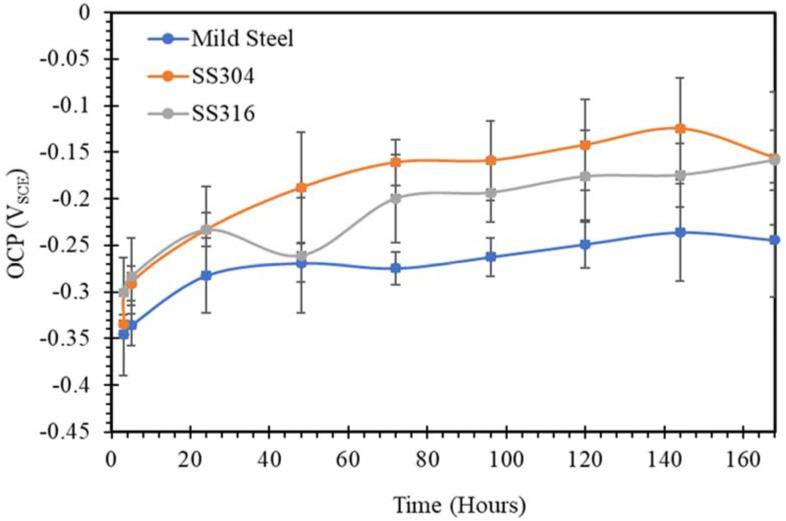
OCP vs. time plots of mild steel, SS304 and SS316 in NC solution.

**Figure 3:**
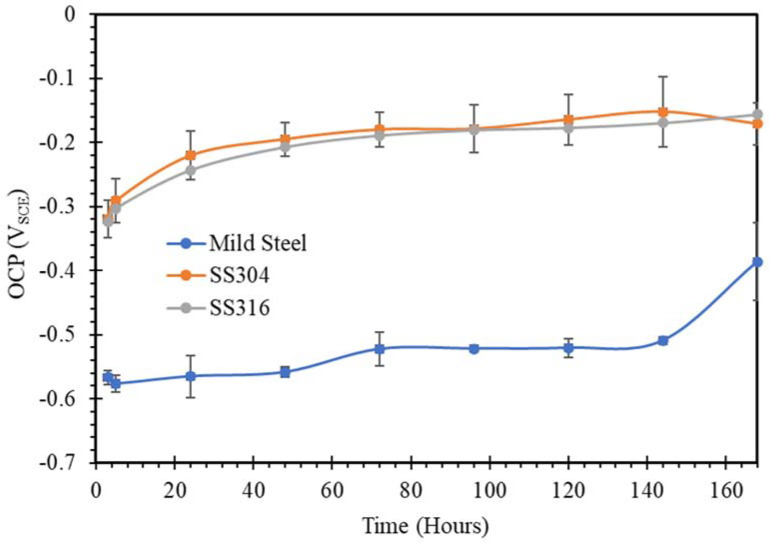
OCP vs. time plots of mild steel, SS304 and SS316 in SWSSNC solution.

**Figure 4:**
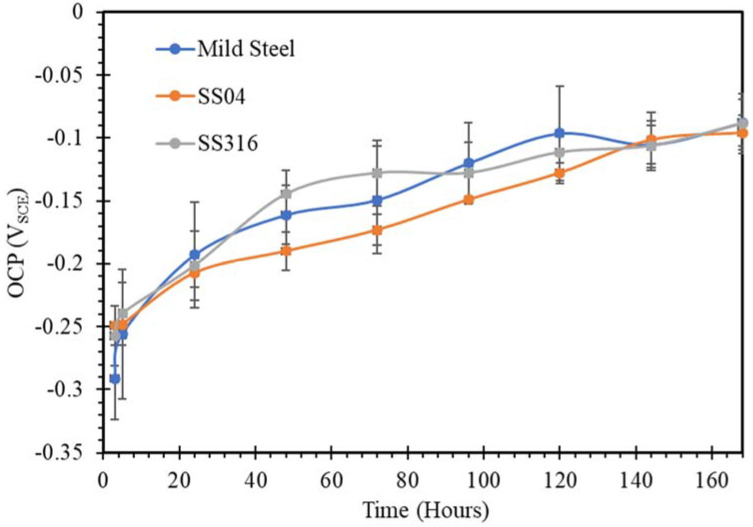
OCP vs. time plots of mild steel, SS304 and SS316 in HPC solution.

**Figure 5:**
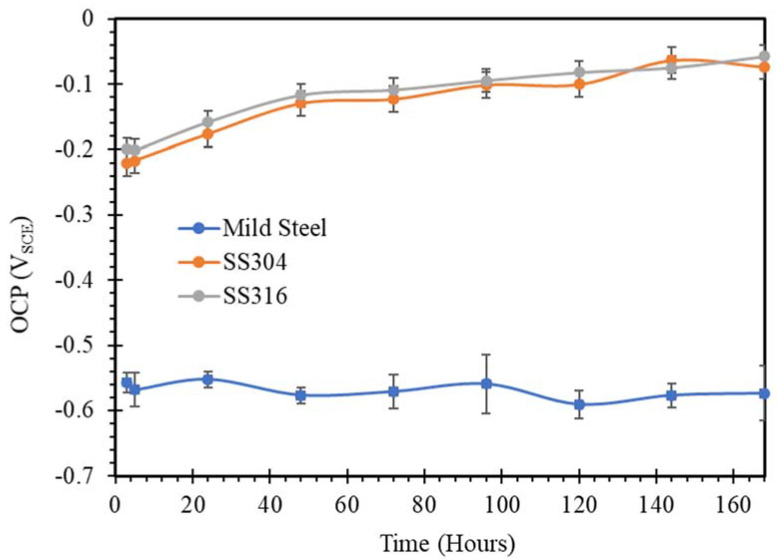
OCP vs. time plots of mild steel, SS304 and SS316 in SWSSHPC solution.

The open circuit potential changing with time for mild steel, SS304 and SS316 in SW, NC, SWSSNC, HPC and SWSSHPC solutions is shown in Figure 1, Figure 2, Figure 3, Figure 4 and Figure 5. In comparing corrosion susceptibility of two metals in a given electrolyte, the metal with more positive OCP suggests its greater thermodynamic stability, and hence, it has lesser susceptibility to corrosion [37]. Therefore, the more the negative OCP of a metal, the greater is its tendency to suffer corrosion.

As expected, when steels were exposed to a simulated SW environment, more positive values of the initial OCP were observed for SS304 and SS316 compared with mild steel (Figure 1). Figure 1 also showed that in 72 h of exposure to a chloride environment, the OCP of samples of both SS304 and SS316 stabilised, remaining reasonably stable during further exposure that was extended to 168 h. It is important to note that the passive layer of chromium oxide that develops on SS in aqueous solutions can be damaged eventually in a solution with sufficient chloride content. However, this did not seem to be happening during the exposure to simulated SW for 168 h. In contrast, the corrosion film of iron oxide/hydroxide that developed on mild steel in an aqueous solution can be quickly damaged when considerable chloride ions are present. As result, OCP of mild steel was considerably more negative, and it continued to be so for the entire test duration, suggesting the continuous formation of the iron oxide/hydroxide film and its disruption due to the high chloride ion content of the environment.

For the two stainless steels (SS304 and SS316) exposed to the alkaline conditions of a normal concrete environment (pH 13.4), OCP values were considerably more negative in an alkaline solution than their OCP values when exposed to an SW environment, as suggested by comparison of data in Figure 1 and Figure 2. However, in a similar comparison, OCP of mild steel was considerably more positive in the alkaline environment. The vast improvement in the corrosion resistance of mild steel in the alkaline environment can easily be attributed to the ability of the steel to form and retain a passive film of iron oxide/hydroxide in such an environment. In fact, in the later stage, the corrosion resistance of mild steel and SS lie in the same regime. Conversely, it is well-known that the passive layer that develops on mild steel in an aqueous environment can be damaged by chloride ions of NaCl, and hence the considerably inferior corrosion resistance of mild steel in SW. However, although an alkaline solution was expected to facilitate formation of a highly passive film of chromium oxide on SS, this passive film may start to get disrupted/dissolved when the pH of the alkaline environment is very high [38], as in the case of NC. Hence, corrosion resistance of stainless steels was adversely affected, as observed from the comparison of OCP data for SS in NC and SW solutions. The disruption of the chromium oxide film on SS by a very high pH alkaline solution may also explain the fluctuations in OCP for SS in the NC solution. However, the passive film continued to provide superior corrosion resistance to SS than did the film in the case of mild steel.

A comparison of OCP in Figure 1 and Figure 3 would suggest the corrosion resistance of mild steel to be similar in the initial stages of immersion in SWSSNC and SW, suggesting the disruption of iron oxide/hydroxide by chloride ions in SWSSNC, and ineffectiveness of its high pH alkaline component. However, with progressing time, the alkaline component began to predominate and corrosion resistance slowly started to improve, as suggested by a gradual positive shift in OCP, and in fact, after about 150 h of immersion, OCP suddenly made a quick positive shift. On the other hand, similar to the behaviour of SS in NC (Figure 2), the effect of the alkaline component of SWSSNC in compromising the effectiveness of the chromium oxide passive film appeared to predominate in the case of SS immersed in SWSSNC, for the entire duration of immersion (Figure 3). As a result, corrosion resistance of SS in SWSSNC was inferior to that in SW, as suggested by the evolution of OCP for SS (Figure 1 and Figure 3).

Ghods et al. [33] found that the same iron oxides (i.e., Fe_3_O_4_/FeO and Fe_2_O_3_/FeOOH) develop on mild steel surfaces exposed to alkaline and alkaline plus chloride environments, though chloride ions decreased the average thickness of the oxide film. The addition of chloride to the alkaline solution changes the stoichiometry of oxide film such that near the film/substrate interface, the relative concentration of Fe^2+^ decreases compared to the Fe^3+^ concentration. The critical amount of chloride in concrete solution for de-passivation of steel depends strongly on the concentration of hydroxides in concrete pore solutions [39,40]. Li and Sagüés [41] noticed that there was an insignificant difference in corrosion resistance of sandblasted steel exposed to an alkaline concrete solution (pH 13.3) when the molar concentration of chloride changed from 0 M in 16 days to 0.8 M at 63 days. In the present study, ~0.6 M was the molar concentration of the chloride in the simulated SWSSC solutions, suggesting that chloride has an insignificant adverse effect on mild steel exposed to SWSSNC, as seen in Figure 3.

The OCP results for immersion of SS and mild steel in HPC and SWSSHPC (Figure 4 and Figure 5) fall well in line with the results and the pertaining explanation for the immersions of the alloys in SWSSNC, NC and SW, as described in the preceding paragraphs. Though the alkali content of HPC was significantly lower (pH 12.7) than that of NC (pH 13.4), the lower content was not expected to change the formation of the corrosion product of iron oxide/hydroxide and resulting passivation of mild steel. Similar to their behaviour in NC, the corrosion resistance of mild steel and SS lie in the same regime also during immersion in HPC (Figure 2 and Figure 4). In contrast, at this lower alkali content of HPC (as compared to NC), the disruption of the chromium oxide protective layer was not as effective. As a result, there were considerably fewer fluctuations in OCP for SS in HPC.

The OCP data and corrosion resistance of SS and mild steel in SWSSHPC were consistent with the earlier discussion of results for HPC, SWSSNC, NC and SW. Thus, the SWSSHPC results further reinforced the explanation for the immersion of results of the alloys in different solutions, as described in the preceding paragraphs.

A comparison of the OCP in Figure 1, Figure 3 and Figure 5 would suggest the corrosion resistance of mild steel to be similar in the initial stages of immersions in SWSSHPC, SWSSNC and SW because of the disruption of iron oxide/hydroxide by chloride ions in each solution, and the inability of the high pH alkaline component to facilitate any effective passivation. However, unlike in the case of SWSSNC where the alkaline component eventually began to predominate (because of the higher alkali content of SWSSNC) and corrosion resistance slowly started to improve (Figure 3), corrosion resistance of mild steel in SWSSHPC continued to be poor throughout the entire immersion period (Figure 5). The reason lies in the lower alkali content of SWSSHPC, which was unable to predominate on the influence of chloride in disrupting the protective layer of iron oxide/hydroxide. Behaviour of SS in SWSSHPC and the pertaining explanation were similar to those for HPC, which reinforced an insignificant effect of chloride in disrupting the chromium oxide protective film during the time span of the immersion test, as was also the case for SWSSNC and SW (Figure 1, Figure 3 and Figure 5).

### 3.2. Potentiodynamic Polarization (PDP)

The PDP results on mild steel and the two SS in the five different concrete environments, as shown in Figure 6, Figure 7 and Figure 8, are examined for the possible interpretations of the PDP results in the light of the discussion on OCP data in Figure 1, Figure 2, Figure 3, Figure 4 and Figure 5.

**Figure 6:**
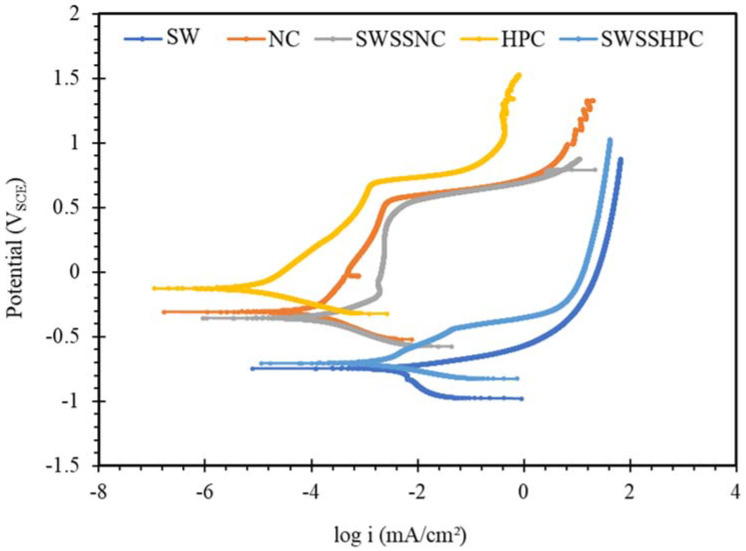
Polarization curves obtained for mild steel exposed to SW, NC, SWSSNC, HPC and SWSSHPC solutions for 168 h.

Discussion on OCP results on mild steel in different solutions concluded that mild steel has the ability to develop a protective corrosion film of iron oxide/hydroxide in the alkaline solutions (without chloride ions), i.e., NC, and HPC, and inability of the steel to develop the protective film in the solution without alkali (i.e., SW). The anodic polarization behaviour of mild steel in these three environments (NC, HPC and SW), as shown in Figure 6, is consistent with the trend in OCP results and the pertaining explanation, i.e., anodic current densities are considerably lower in the alkaline solutions (without chloride ions), i.e., NC, and HPC, and that in SW is considerably higher. For instance, the current densities of mild steel in NC and HPC solutions are lower by more than 1.5 orders of magnitude and 3 orders of magnitude, respectively, than that in SW. In the potential range of ~0.25–0.7 V_SCE_ above OCP, the minimal change in current density with an increase in anodic potential demonstrated/confirmed the ability of the steel to form a robust passive film in NC and HPC upon increase in the anodic potential. In contrast, in SW, the current density continued to increase sharply with increasing anodic potential above OCP because the chloride ions did not allow formation of any stable passive film. PDP results in SWSSNC and SWSSHPC were also consistent with the corresponding OCP results. It was concluded from the OCP results that the higher alkali content of SWSSNC (than SWSSHPC) predominated the detrimental effect of chloride and allowed formation of a protective/passive layer of iron oxide/hydroxide, whereas such a protective layer could not form in SWSSHPC (because of its lower alkali content). Accordingly, similar to the behaviour in NC, minimal change in current density was observed in SWSSNC with an increase in anodic potential above OCP (in the range of −0.35 to ~0.7 V_SCE_), whereas in SWSSHPC, the current density continued to increase with increasing anodic potential above OCP (i.e., similar to behaviour in NaCl), as shown in Figure 6. However, it was not clear why the corrosion resistance was somewhat superior in HPC (which has lower alkali content) than in NC or SWSSNC.

**Figure 7:**
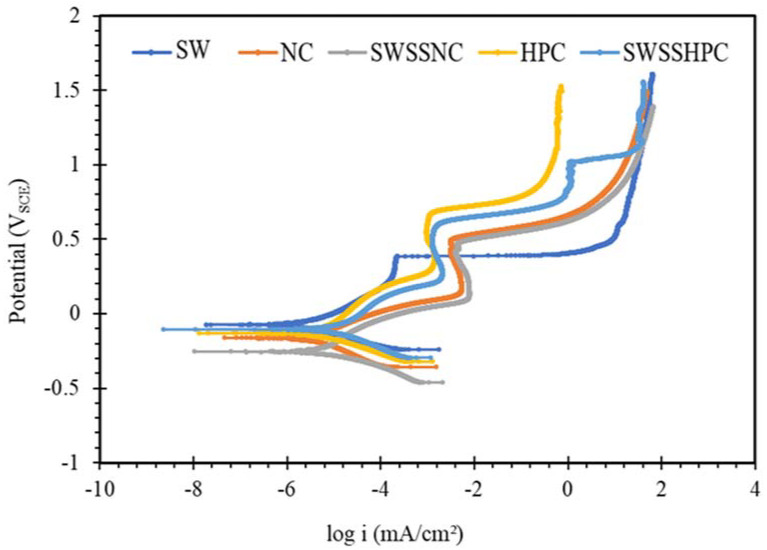
Polarization curves obtained for SS304 steel exposed to SW, NC, SWSSNC, HPC and SWSSHPC solutions for 168 h.

**Figure 8:**
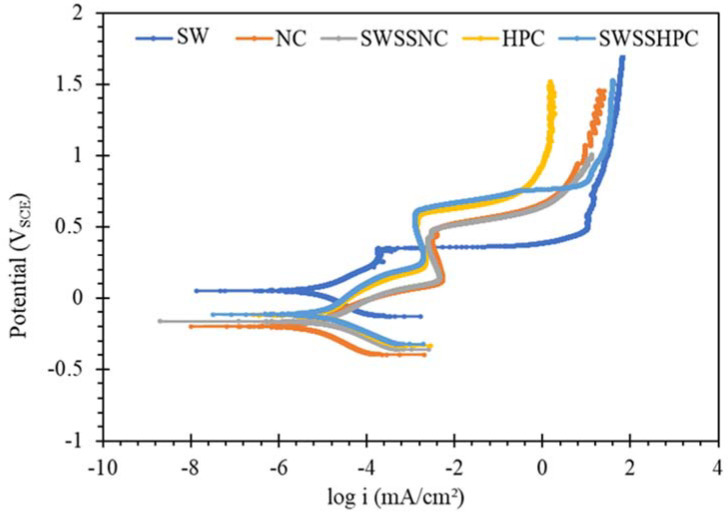
Polarization curves obtained for SS316 exposed to SW, NC, SWSSNC, HPC and SWSSHPC solutions for 168 h.

PDP results for SS in different solutions (Figure 7 and Figure 8) were also consistent with the trend in OCP data in the corresponding solutions (Figure 1, Figure 2, Figure 3, Figure 4 and Figure 5). OCP results showed the tendency of SS to passivate in aqueous solutions, including in NaCl solutions, but the passive films of chromium oxide were disrupted by high pH (>13) alkali solutions (NC and SWSSNC), whereas the passive films were more stable in the solutions with lower alkali content (HPC and SWSSHPC). Additionally, during PDP tests, SS in NaCl showed lower current density than those exposed to HPC, SWSSNC and SWSSHPC and signs of passivation upon increase in anodic potential to 0.4 V_SCE_, when the passivity suddenly collapsed leading to sudden increase in current density (obviously due to disruption of the passivity by chloride ions). In each of the solutions containing alkali, a well-established regime of distinct passivity was observed that continued over a considerable range of increase in anodic potential, over which the anodic current densities, in fact, decreased with increasing potential. A further increase in the anodic potential caused disruption in the passive film and a sudden increase in current density. Interestingly, again consistent with the OCP data, the passive films were more robust, and corresponding critical current densities (i_crit_) were lower in the solutions of lower alkalinity (pH 12.7 as in HPC and SWSSHPC) than in the solutions with higher alkalinity (pH 13.4, as in NC and SWSSNC). As discussed in the context of OCP data, the higher pH disrupted the passive film of chromium oxide that develops on SS. This explanation was supported by the E_pit_ data, as it took a higher anodic potential to cause pitting in solutions of lower alkalinity (pH 12.7). Note, E_pit_ is the potential at which the passive film starts to get disrupted that causes pitting.

### 3.3. Electrochemical Impedance Spectroscopy (EIS)

**Figure 9:**
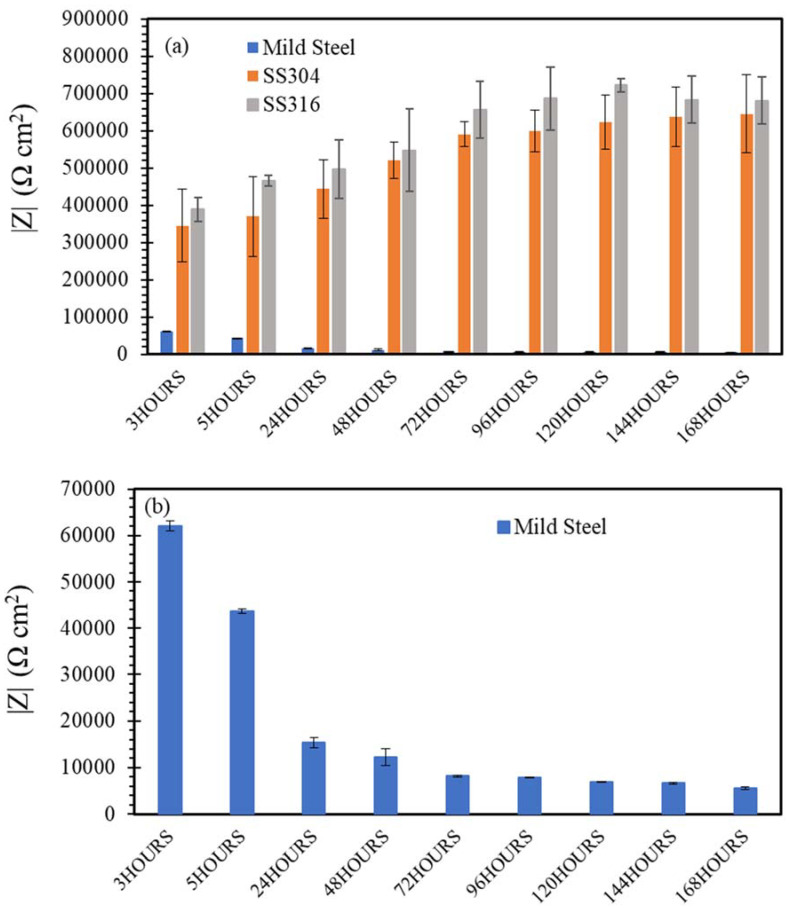
(**a**,**b**) Evolution of impedance |Z| for of mild steel, SS304 and SS316 in SW solution.

**Figure 10:**
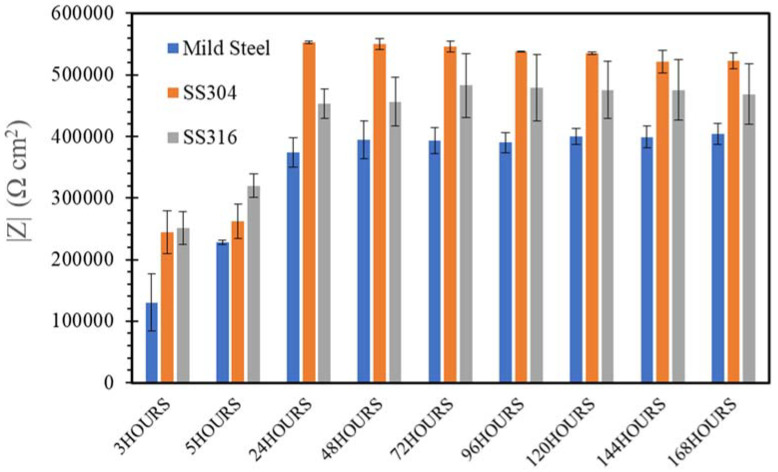
Evolution of impedance |Z| for of mild steel, SS304 and SS316 in NC solution.

**Figure 11:**
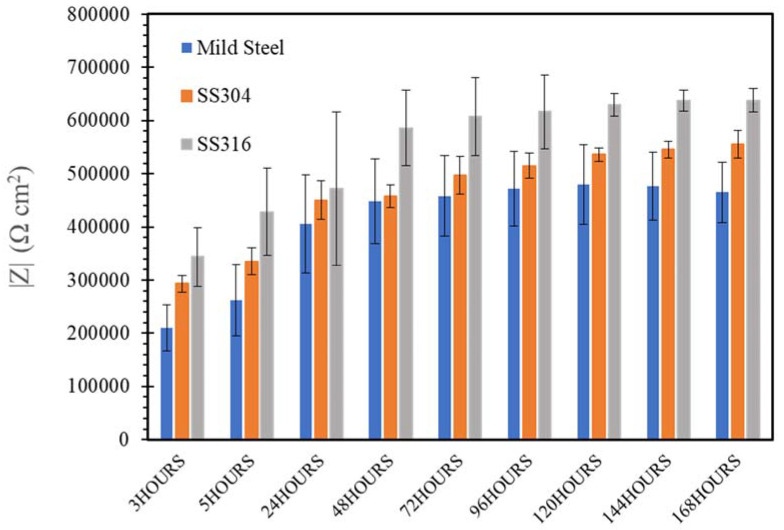
Evolution of impedance |Z| for of mild steel, SS304 and SS316 in HPC solution.

**Figure 12:**
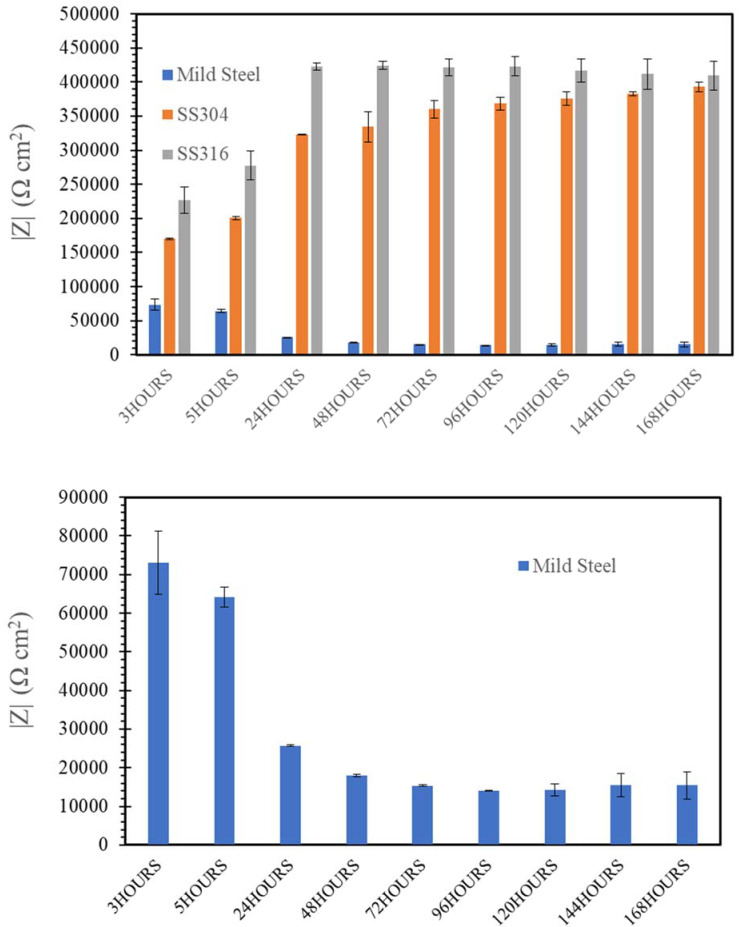
Evolution of impedance |Z| for of mild steel, SS304 and SS316 in SWSSNC solution.

**Figure 13:**
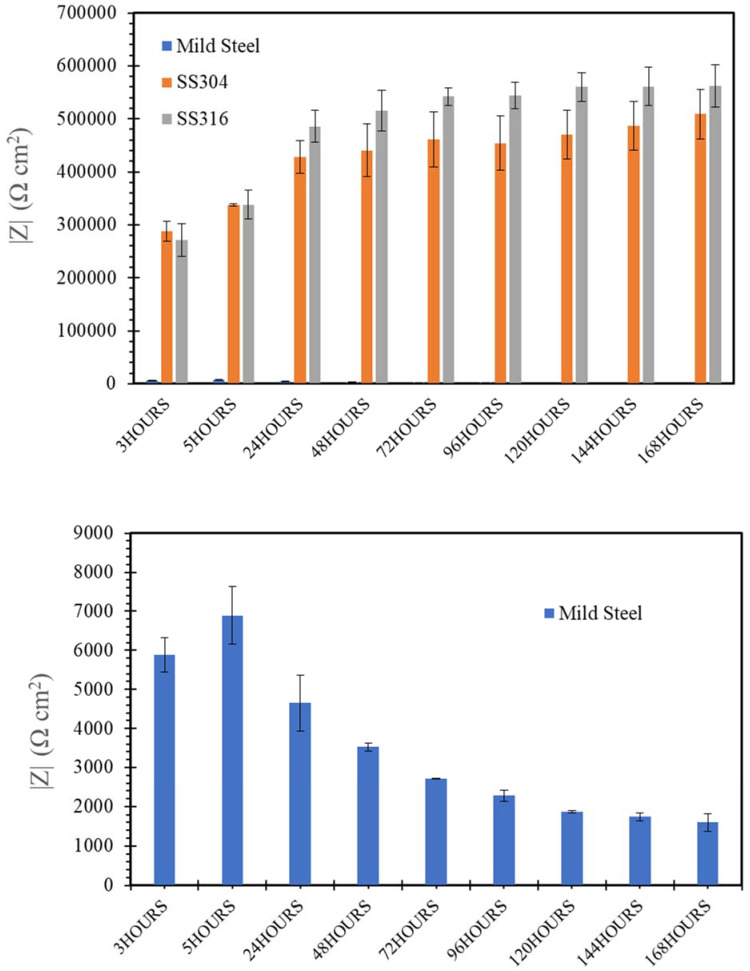
Evolution of impedance |Z| for of mild steel, SS304 and SS316 in SWSSHPC solution.

Figure 9, Figure 10, Figure 11, Figure 12 and Figure 13 depict the impedances of mild steel and stainless steels exposed to the simulated concrete and SW solutions. Typical Bode plots for mild steel and stainless steels exposed to a typical simulated environment and the electrical equivalent circuits (EECs) that fit the data are shown in Appendix A.

When exposed to a SW environment (Figure 9), mild steel showed much lower impedance values compared to stainless steels. Corrosion resistance of the mild steel stabilized in 48 h of exposure to the NaCl solution, and then remained reasonably stable for the rest of the exposure, i.e., up to 168 h (Figure 9b). However, the impedance values of stainless steels, pre-exposed to a chloride solution for 48 h, were more than 11 times greater than that of mild steel pre-exposed for the same duration. The difference in the impedance continued to increase slowly and reached than 113 times higher than that of mild steel after 168 h of immersion.

Although the chloride in the SW solution had a detrimental effect on corrosion resistance of mild steel, it had little effect on SS. Chromium (Cr) in SS played a crucial role in the corrosion resistance of SS by forming a strong and more stable passive film on stainless steel to enhance corrosion resistance in the NaCl solution. It was also observed that SS316 had higher impedance compared with SS304 because the addition of molybdenum (Mo) in SS316 improved its pitting corrosion resistance and enhanced the protectiveness of the passive film due to the Mo^−^ and Cr^−^ enriched film formation. In addition, it was suggested that molybdate ions (MoO_4_^2−^) can form upon dissolution of Mo^−^, which effectively blocks the adsorption of chloride ions (Cl^−^) [42]. These results were consistent with the results of OCP and PDP, demonstrating the superior corrosion resistance of SS316 among the three steels when exposed to a chloride environment.

Figure 10 and Figure 11 display the impedance values of the steels pre-exposed to the alkaline environments (NC and HPC) for 168 h. The impedances of mild steel are considerably higher with little variation over time (i.e., during the immersions for 3–168 h), when compared with exposure to the SW environment (Figure 9). As noted earlier, the formation of iron oxide/hydroxide film on the mild steel surface in the alkaline environment improved its corrosion resistance, which is consistent with the OCP and PDP results of mild steel exposed to an alkaline environment.

Although the corrosion resistance of the two SS improved with increasing time of immersion in NC, SS304 showed somewhat superior resistance compared to SS316. Such behaviour could be attributed to the somewhat higher Cr content of SS304 (i.e., 18.300 wt.%) compared to SS316 (Cr, 16.800 wt.%) as seen in Table 1, because the surface layer of Cr-oxide in SS is susceptible to dissolution by highly alkaline environments such as NC. This is consistent with the findings of Liu et al. [43].

In contrast, for the two SS, the impedance is higher when exposed to HPC (pH = 12.7), as compared with SS316 when exposed to NC (pH = 13.4), which indicated that the passive film formed on the SS surface in NC was not as robust as the one that formed in HPC. This proved again the conclusion of OCP and PDP results that the corrosion resistance of stainless steels is adversely affected by the very high level of alkalinity of NC. The adverse effect of high alkalinity on steel corrosion seemed to be more apparent in SS316, which may be because the high alkalinity seriously affected the Mo- and Cr-enriched film formation.

Figure 12 and Figure 13 showed that the presence of Cl^−^ ions in seawater sea sand concrete solutions (SWSSHPC or SWSSNC) had little effect on corrosion resistance of SS during exposure up to 168 h. Both SS possessed corrosion resistance similar to that observed during immersion in NC and HP during exposure time within 24 h. The impedance of both SS was higher when exposed to SWSSHPC compared with SWSSNC, which is again consistent with the OCP and PDP results. This indicates that the high alkalinity played a more predominant role in reducing the corrosion resistance and chloride seemed to have insignificant effect on corrosion resistance of SS in the SWSSC environment. Additionally, similar to OCP and PDP results, EIS data also suggest that an increase in alkalinity improves the corrosion resistance of mild steels in alkaline solutions with chloride content. EIS data suggest a continuous decrease in impedance of mild steel with increasing immersion time for 168 h in SWSSHPC (which has much lower alkali content compared with SWWSSNC). This indicated that the higher alkali content of SWSSNC can allow the formation of a more robust passive layer on mild steel that resisted the disruption of the film by chloride ions. In the case of SWSSNC, after an initial large decrease in corrosion resistance (during the first 48 h) caused by chloride, the corrosion rate (impedance) started to gradually improve for the rest of the exposure time, as a result of the ability of the high alkali content to repair the damage caused to the passive layer by the chloride ions, which is consistent with the OCP results.

### 3.4. Corrosion Morphology

**Figure 14:**
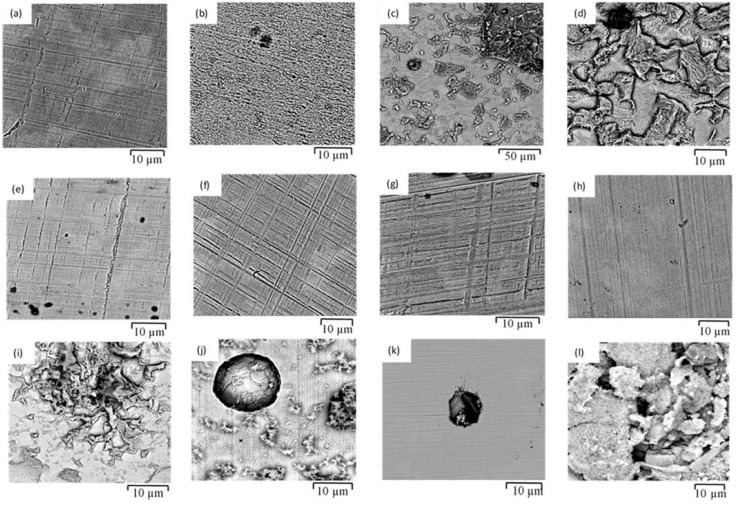
SEM micrographs of morphology of mild steel exposed to different simulated solutions of: (**a**,**b**) SW for 3 h, (**c**) SW for 72 h, (**d**) SW for 168 h, (**e**) NC for 168 h, (**f**) HPC for 168 h, (**g**) SWSSNC for 168 h, (**h**,**i**) SWSSHPC for 3 h, (**j**) SWSSHPC for 72 h and (**k**,**l**) SWSSHPC for 168 h.

Figure 14a–d show the SEM images of mild steel exposed to SW after 3, 72 and 168 h. For 3 h exposure to SW, two apparently distinct areas were observed on the steel surface, i.e., the areas with plain and rough features (Figure 14a,b, respectively). Due to the attack by Cl^−^ ions, the mild steel surface started to become uneven, with some corroded and rough features. As it is difficult for mild steel to sustain a passive film in seawater, the overall surface becomes corroded and corrosion product is deposited in 72 h (Figure 14c). The steel surface becomes extensively corroded in 168 h, as shown in Figure 14d. In contrast, such features of heavy corrosion were absent for the mild steel surface exposed to the simulated solutions of NC and HPC for 168 h (Figure 14e,f, respectively), as a uniform passive film developed due to the highly alkaline environment; the surface topographic features are attributed solely to the grinding marks. It is reported [33,40] that the oxide film developed on carbon steel in simulated concrete solution (pH 13.3) is relatively uniform, and there is no remarkable differences in the characteristics of the oxide across the steel surface.

As suggested in the description of the electrochemical tests, the highly passivating influence of the high alkalinity predominated the deleterious influence of chloride content in SWSSNC. As a result, the mild steel exposed to SWSSNC for 168 h (Figure 14g) bears a uniform passive film on the entire surface, i.e., similar to those observed for mild steel in NC for 168 h (Figure 14e). In fact, the major fraction of the steel surface exposed to SWSSHPC for 3 h developed a uniform passive film (Figure 14h); however, localised attack was observed in some areas (Figure 14i), which is attributed to a chloride attack. The fraction of surface area with such attack as well as the depth of such attack increased with increasing exposure time in SWSSHPC (Figure 14j–l).

**Figure 15:**
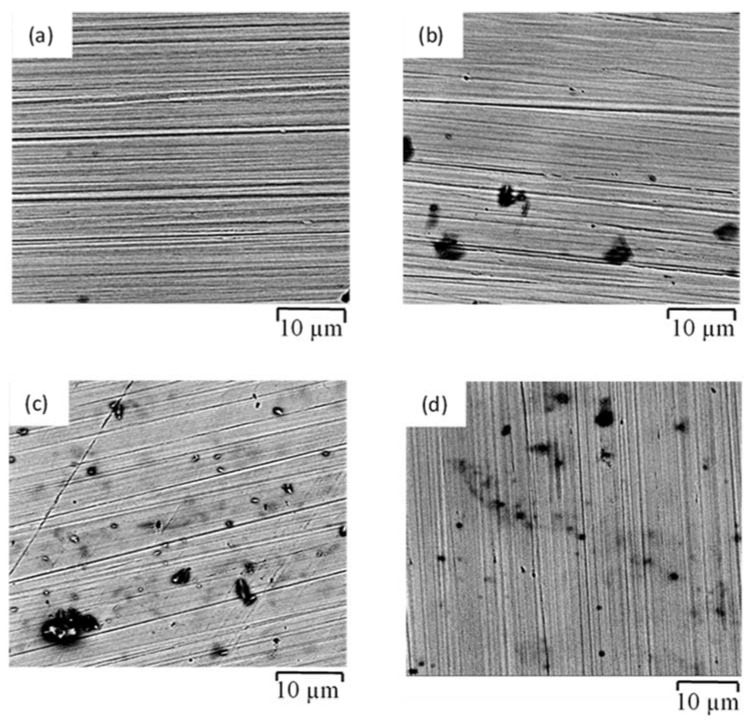
SEM images of morphology SS316 exposed to different simulated solutions of: (**a**) SW for 168 h, (**b**) NC for 168 h, (**c**) SWSSNC 72 h and (**d**) SWSSHPC 72 h.

Figure 15 summarizes the main morphological observations of 316SS exposed to the simulated solutions of SW, NC, SWSSNC and SWSSHPC. Compared with mild steel, the SEM image in Figure 15a of SS316 exposed to SW indicates that Cl^−^ has no apparent effect in 168 h of exposure to SW, which is attributed to the formation of a robust passive film due to sufficient Cr content of the steel that resists the aggressive Cl^−^ environment. This is consistent with the EIS and PDP results. However, the exposure to alkaline solution could compromise the passive film robustness as suggested by electrochemical tests, which are supported by SEM images showing the dark spots due to localised attack that started to develop in 168 h of exposure to NC, and 72 h to SWSSNC and SWSSHPC solutions, as seen respectively in Figure 15b–d.

## 4. Conclusions

The electrochemical degradation behaviour of a mild steel and two stainless steels, SS304 and SS316, exposed for a maximum duration of 168 h to various simulated solutions of sea water (SW), normal concrete (NC), seawater sea sand concrete (SWSSNC) and seawater sea sand high performance concrete (SWSSHPC) was investigated. The following conclusions were drawn:

1. SW has an inimical effect on corrosion resistance of mild steel, whereas it has little impact on stainless steels (SS316 and SS304).
2. The corrosion resistances of mild steel are similar in the initial stages of immersions in the simulated solutions of SWSSHPC, SWSSNC and SW because chloride content of each solution was able to disrupt the protective layer of iron oxide/hydroxide. However, as time progressed, the corrosion resistance slowly started to improve in the SWSSNC solution that had high alkali content, as also demonstrated through SEM imaging, whereas corrosion resistance was compromised considerably in SWSSHPC and SW.
3. The corrosion resistance of SS is compromised in the highly alkaline environment as suggested by electrochemical results. Post-corrosion morphologies support this finding, as the dark spots started to develop on steel surfaces due to localised attack in 168 h of exposure to NC, and in 72 h to SWSSNC and SWSSHPC solutions.

## Figures and Tables

**Table 1:** Chemical composition of steels used in the present study.

Steel Type	Chemical Composition (wt.%)											
	C	Mn	Si	S	P	Cr	Ni	Cu	Mo	Co	N	Al
SS316	0.027	1.800	0.360	0.026	0.038	16.800	10.060	0.450	2.030	0.190	0.075	
SS304	0.021	1.580	0.290	0.021	0.038	18.300	8.0300	0.520	0.360	0.170	0.078	
Mild steel	0.140	1.220	0.200	0.025	0.022	0.030	0.010	0.008	0.002	-	-	0.005

**Table 2:** Chemical composition and pH of test solutions.

Simulated Solution	Quantities (g/L)				pH
	NaOH	KOH	Ca(OH)_2_	NaCl	
NC ^a^	2.4	19.6	2		13.4
SWSSNC ^b^	2.4	19.6	2	35	13.4
HPC ^a^	0.6	1.4	0.037		12.7
SWSSHPC ^b^	0.6	1.4	0.037	35	12.7
SW				35	7.5

^a^ Simulated solutions of NC and HPC are prepared according to [34]. ^b^ Simulated solutions of SWSSNC and SWSSHPC were prepared according to [4,15,35,36].

**Figure A1:**
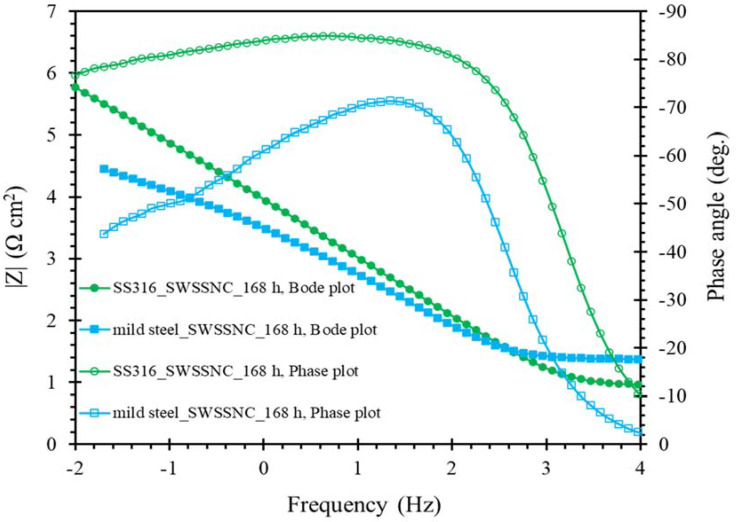
Bode plots and phase plots for mild steel and SS316 pre-immersed in SWSSNC for 168 h.

**Figure A2:**
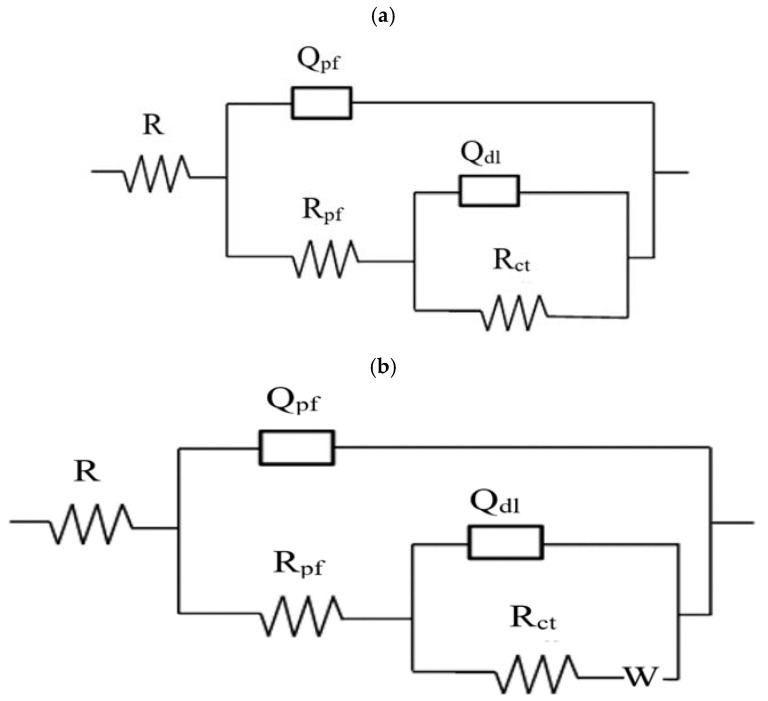
Equivalent electrical circuits that fit the impedance data for the steels pre-exposed to SWSSNC for various durations: (**a**,**b**) for mild steel, and (**b**) stainless steels.

## Data Availability

Data are contained within the article.
